# Time-Restricted Eating and Prebiotic Supplementation Demonstrate Feasibility and Acceptability in Young Adult Pediatric Cancer Survivors: A Randomized Controlled Pilot Trial

**DOI:** 10.3390/nu17203306

**Published:** 2025-10-21

**Authors:** Kate Cares, Manoela Lima Oliveira, Alyssa Bryner, Bernice Man, Zhengjia Chen, Beatriz Peñalver Bernabé, Mary Lou Schmidt, Marian Fitzgibbon, Kelsey Gabel

**Affiliations:** 1 Department of Kinesiology and Nutrition, University of Illinois Chicago, Chicago, IL 60612, USA; abryne2@uic.edu (A.B.); kdipma2@uic.edu (K.G.); 2 University of Illinois Cancer Center, University of Illinois Chicago, Chicago, IL 60612, USA; znchen@uic.edu (Z.C.); mlf@uic.edu (M.F.); 3 Department of Health and Human Physiology, University of Iowa, Iowa City, IA 52242, USA; manoela-limaoliveira@uiowa.edu; 4 Department of Medicine, Division of Academic Internal Medicine and Geriatrics, University of Illinois Chicago, Chicago, IL 60612, USA; bernicem@uic.edu; 5 Department of Biomedical Engineering, College of Medicine, University of Illinois Chicago, Chicago, IL 60612, USA; penalver@uic.edu; 6 Department of Pediatrics, University of Illinois Chicago, Chicago, IL 60612, USA; mls3@uic.edu

**Keywords:** time-restricted eating, prebiotics, pediatric cancer survivors

## Abstract

Background: The optimization of treatment for pediatric cancer has increased 5-year survivor rates to over 80%. Currently, there are almost half a million survivors of a pediatric cancer alive in the United States, with numbers increasing worldwide. Despite increased survivorship, pediatric cancer survivors (PCSs) are at high risk for long-term chronic disease, including cardiometabolic dysregulation at an early age due to cancer-related treatments. PCSs often have increased adiposity, perturbation in the gut microbiome, and chronic systemic inflammation compared to age-matched controls. Time-restricted eating (TRE) has emerged as an effective dietary intervention to promote weight loss in individuals with increased adiposity and cardiometabolic disease. Prebiotic supplements may enhance the efficacy of TRE by promoting satiety via the gut microbiome. Given the accessibility of both TRE and prebiotic supplements, this type of dietary intervention may be ideal for young adult PCSs. The purpose of this study was to determine the feasibility and acceptability of 12 weeks of TRE with and without a prebiotic supplement among young adult PCSs. Changes in body weight, body composition, and cardiometabolic disease risk markers were explored. Methods: Feasibility was measured based on recruitment (*n* = 20), retention (>80%), and adherence to the TRE eating window and prebiotic (>80%), and acceptability was measured based on a validated survey. Body weight, body composition, blood pressure, and additional blood-based cardiometabolic disease risk markers were also measured before and following the intervention. Results: Feasibility was not met based on recruitment (*n* = 13), but retention and adherence exceeded the a priori hypothesis. Acceptability also met the a priori hypothesis. Improvements were observed in some cardiometabolic disease risk markers, including a significant decrease in fat mass and visceral fat mass in both groups following the intervention. Conclusions: Given the positive outcomes related to retention, adherence, and acceptability, as well as some cardiometabolic disease risk markers, a larger and longer study of TRE and prebiotic supplementation in PCSs is warranted. However, innovative recruitment strategies should be implemented, such as leveraging social media and targeting larger geographical areas, given recruitment challenges.

## 1. Introduction

Pediatric cancer incidence rates have been increasing approximately half a percent each year since 1975, and cancer-related mortality remains one of the leading causes of death among individuals younger than 18 years of age [1]. Notably, advances in cancer treatment over the past several decades have resulted in an increase in survivorship, particularly within pediatric oncology. The 5-year survival rate among pediatric cancer survivors (PCSs) is over 84%. There are over 420,000 PCSs identified within the United States [2] and a projected increase to 26.1 million PCSs worldwide by 2040 [1]. Despite the evolution of cancer therapy, PCSs experience higher rates of obesity, hypertension, dyslipidemia, and insulin resistance as compared to the general population [3,4,5,6,7,8,9], leaving this population vulnerable to increased risk for chronic diseases. Improving diet and reducing obesity has the potential to improve cardiometabolic disease risk and reduce the chronic disease burden among PCSs and improve long-term health and quality of life.

During treatment, pediatric cancer patients are encouraged to consume calorie-dense, highly palatable foods to prevent malnutrition [10] and are often sedentary due to fatigue and muscle weakness [11]. These behavioral practices are often carried over into survivorship, as shown in larger observational studies of diet and lifestyle in this population [12,13,14,15,16]. PCSs face significant barriers to lifestyle behavior change due to stress and fatigue, and few intervention studies have targeted weight management or cardiometabolic disease prevention in this population [17].

Time-restricted eating (TRE) has emerged as an alternative to calorie restriction for weight management where individuals watch the clock as opposed to counting calories or tracking foods. TRE comes without the burden of calorie counting or purchasing unfamiliar or expensive foods. Accumulating evidence suggests that TRE produces a natural calorie deficit of approximately 300–500 calories per day, based on self-report, resulting in weight loss in an 8–12-week period [18]. Reductions in visceral body fat, waist circumference, and blood pressure, and improvements in glucose homeostasis have also been observed following a TRE regimen in the non-survivor population [19,20,21,22,23]. Given its salient features, TRE is highly accessible to all ages and socioeconomic groups. Despite the ease of this regimen, it has yet to consistently promote clinically significant weight loss of 5% or greater [24]. Additionally, few studies have found significant changes in gut microbiome composition and metabolic activity following TRE [25,26], which may be due to insignificant changes in diet quality under this regimen.

Studies suggest that the microbes in an individual’s gut can beneficially modulate body weight, food intake, glucose homeostasis, and insulin sensitivity [27]. Short-chain fatty acids (SCFAs) are metabolites produced by microbial fermentation of complex carbohydrates, including dietary fibers [28]. Prebiotics are substrates that are selectively fermented by gut microbes that can shift the composition and function of the gut microbiome and confer health benefits to the host [29]. Galacto-oligosaccharides (GOS) are a type of prebiotic supplement that can be effective in increasing the abundance of SCFA-producing microbes with downstream positive effects on the host, including increased satiety and decreased inflammation among adults with obesity [30,31,32,33]. Because TRE alone does not provide the fiber substrate needed for gut microbial changes, combining a prebiotic supplement (i.e., GOS) with TRE could have a greater cumulative impact on body weight and cardiometabolic disease risk markers than TRE alone.

The purpose of this pilot study was to determine the feasibility, acceptability, and safety of 12 weeks of TRE with and without a prebiotic supplement among young adult PCSs. Changes in body weight, body composition, glucose regulation, and cardiometabolic disease risk markers were explored.

We hypothesized that the study would be feasible with ≥30% enrollment of young adult PCSs that were screened as eligible via medical record data. Feasibility would be measured by participants completing more than 80% of planned study visits and retention of ≥ 80% of participants in the two study groups, which include an 8-h TRE group and an 8-h TRE with a prebiotic supplement. It was anticipated that the adherence to TRE would be 80% or higher throughout the intervention for both TRE groups and that TRE and TRE with prebiotic would be safe with no significant intervention-related adverse events throughout the intervention.

This study fills a critical gap in knowledge essential for minimizing morbidity in PCSs. Aiming to reduce cardiometabolic disease risk factors with low-maintenance interventions has the potential to improve longevity and quality of life in this high-risk population.

## 2. Methods

The following protocol was approved by the University of Illinois Chicago Institutional Review Board (IRB #2022-0745) on 20 February 2023. Participants provided written informed consent prior to study participation. The trial was registered at ClinicalTrials.gov (NCT05826184) on 21 April 2023.

### 2.1. Study Design (Figure 1)

A 12-week randomized controlled pilot trial was conducted among young adult PCSs with overweight or obesity who had a diagnosis of a pediatric cancer defined by the International Classification of Childhood Cancer (ICCC) [34]. The ICCC classifies pediatric cancer based on the histological characteristics and primary site of the tumor, which include but are not limited to many leukemias, lymphomas, central nervous system neoplasms, neuroblastomas, retinoblastomas, and sarcomas [34]. Participants were randomized 1:1 to one of two groups: (1) 8-h TRE, ad libitum eating between 12:00 PM and 8:00 PM, and water fasting from 8:00 PM to 12:00 PM daily, or (2) 8-h TRE, plus a commercially available prebiotic supplement (PreTRE; 3.65-g sachet of Bimuno^®^ GOS; 2.75 g active GOS; Clasado Ltd., Reading, UK). No control group was deemed warranted, as this is a pilot study targeting a vulnerable group in which an intervention is justified for all participants. Additionally, this is a challenging population to recruit, given that other behavioral interventions in PCSs have had challenges meeting recruitment goals and high attrition rates [35,36,37,38].

### 2.2. Setting, Screening, and Recruitment

Young adult PCSs from the Childhood Cancer Survivorship Clinic (CCSC) at the University of Illinois Chicago (UIC) were screened via the University of Illinois Health and Hospital system electronic record system or through in-person enrollment in the UIC Childhood Cancer Survivorship Clinic. Collaboration between the study team and clinical care team assured access to the appropriate patient population and provided medical oversight for the pilot trial. Additionally, participants were recruited via postcards, posting on social media, emails through listservs, and at survivorship events. Potential participants filled out a remote screener distributed by email and collected electronically using REDCap ^TM^ (Research Electronic Data Capture, 2201 Vanderbilt Place, Nashville, TN, USA) [39] to determine preliminary eligibility and interest.

### 2.3. Participants

#### 2.3.1. Inclusion

Participants were between the ages of 18 and 39 years old, had completed anti-tumor treatment for pediatric cancer, and had a BMI within 25.00–49.99 kg/m^2^. The upper limit for BMI was included due to limitations on body size for the DXA scan. All participants were able to provide written informed consent and had the ability to understand and comply with study procedures.

#### 2.3.2. Exclusion

Exclusion criteria included individuals on insulin, BMI ≥ 50 kg/m^2^ and <25 kg/m^2^, pregnant, trying to become pregnant or breastfeeding (a negative serum or urine pregnancy test was required per institutional practice guidelines at screening as well as prior to all dual-energy X-ray absorptiometry (DXA) scans), shift workers who maintain a work schedule that crosses 12:00 AM on >1 day per week, a history of eating disorders, active infection requiring systemic therapy, illicit drug use (excluding self-reported marijuana), excessive use of alcohol (i.e., >2 drinks/day), currently participating in a structured weight loss program, >3% weight loss in three months prior to recruitment, already adhering to an average eating window <10 h per day based on self-report, history of myocardial infarction, stroke, congestive heart failure, chronic hepatitis, cirrhosis, or chronic pancreatitis, solid organ transplantation, lack of access to the Internet, antibiotic use within 2 months prior to the initiation of the study, frequent and regular use (>3 times per week) of prebiotics, probiotics, synbiotics or laxatives, uncontrolled HIV/AIDS or active viral hepatitis, any mental or medical condition that prevented the patient from giving informed consent or participating in the trial, or any other major comorbidity determined by the study team, were excluded.

#### 2.3.3. Randomization

Randomization and allocation to the study group were conducted using sealed opaque envelopes prepared by an individual outside of the data collection team following baseline data collection. The small size of the study warranted this type of randomization due to the practicality of the method and the inability to use a stratified method due to the limited number of participants. Participants were randomized in a 1:1 ratio to one of two groups: (1) TRE or (2) TRE and GOS (PreTRE).

### 2.4. Interventions

#### 2.4.1. TRE—8-h Time-Restricted Eating (12 Weeks)

Participants randomized to this group were instructed to follow the 8-h TRE protocol. This protocol consisted of instructions to consume food ad libitum (no calorie or food restrictions) during the hours of 12:00 PM and 8:00 PM daily. Calorie-free drinks such as water and black coffee were permitted during the fasting period, 8:00 PM–12:00 PM. Qualitative data among this age group indicates that this population may prefer a later eating window [40,41], and observational data has reported 800,000 North Americans usually place their TRE window from 12:00 to 8:00 pm [42]. Given the nature of this pilot study, a later eating window was selected to ensure robust recruitment, retention, and compliance outcomes.

Each participant received an education packet at baseline containing guidelines for TRE and was given verbal instructions with time to answer any questions. A registered dietitian that was part of the research team met virtually with the TRE participants on a weekly basis. During weekly appointments the registered dietitian assessed body weight (measured remotely by the participants), TRE adherence (self-reported), discussed any questions regarding the intervention, and completed an adverse event-related questionnaire.

#### 2.4.2. PreTRE—8-h Time-Restricted Eating Plus Prebiotic Supplement (12 Weeks)

Participants randomized to this group followed the protocol for the 8-h TRE intervention with the addition of a prebiotic supplement. Participants consumed 3.65 g (standard daily dose) of a GOS-based prebiotic supplement (Bimuno^®^ GOS; Clasado Ltd.; Reading, UK) mixed with 8 oz of food or drink of choice once daily with their first meal. Participants in the PreTRE group met virtually with a registered dietitian weekly, following the same protocol.

#### 2.4.3. Participant Compensation and Accommodation

Each subject received a Fitbit Inspire 3^TM^ (Google LLC, Mountain View, CA, USA) that was to be used throughout the intervention and a monetary subsidy for participating in the trial. Outside of the baseline and week 12 outcome measurements, the intervention sessions were 100% remote, with multiple time offerings per week to accommodate commute delays and scheduling and decrease participant burden. Additionally, no dietary restrictions were required on major holidays (i.e., Thanksgiving or Christmas Day).

### 2.5. Data Collection and Measurements

All measures were obtained at baseline and following the 12-week intervention period. Data collection took place at the Human Nutrition Research Unit at the College of Applied Health Sciences, University of Illinois Chicago.

#### 2.5.1. Feasibility

We collected detailed records of the number of individuals eligible, approached, and screened and the number of individuals who declined and their reason(s) for non-enrollment. Loss to follow-up/withdrawal was closely monitored. To track the progress of participants through the trial, we adhered to and updated weekly the Consolidated Standards of Reporting Trials (CONSORT) [43] participant flow diagram. Individuals who voluntarily withdrew were asked to provide reason(s) for revoking their enrollment.

Attendance at study visits, completeness of data, intervention session attendance, and self-reported adherence to the eating window and prebiotic ingestion were also assessed. TRE and PreTRE adherence were monitored by patient-reported start and stop times of food intake daily (via a secure text message), with adherence defined as up to 30 min before 12:00 PM and up to 30 min past 8:00 PM. An additional TRE adherence metric included adherence to an eating window of ≤8.5 h (510 min) per day. In addition to following the TRE protocol, participants in the PreTRE group were asked to save all sachets from the supplements to confirm the number of days the prebiotic was consumed.

#### 2.5.2. Acceptability

Participants in both groups completed a Diet Satisfaction Survey at baseline and post-intervention. This survey consisted of seven domains: Healthy Lifestyle, Convenience, Cost, Family Dynamics, Preoccupation with Food, Negative Aspects, and Planning and Preparation [44]. Each domain consisted of several questions with an aggregate score from 1 to 5, where lower scores reflected “disagreement,” a score of “3” reflected a neutral stance, and higher scores reflected “agreement.” The “healthy lifestyle” domain consisted of 8 questions related to how an individual might feel the diet they are currently engaged with reflects a “healthy lifestyle.” The “convenience” domain consisted of 9 questions related to how easily a participant could find food to fit the diet with which they were engaged. The “cost” domain consisted of 5 questions related to how easily the diet could be followed based on the participant’s financial capacity. The “family dynamics” domain consisted of 6 questions related to the perceived support the participant was receiving from friends and family regarding following the diet they were currently engaged with. The “preoccupation with food” domain consisted of 6 questions related to the degree to which a participant did not experience chronic hunger while following the diet they were currently engaged with. The “negative aspects” domain consisted of 6 questions related to the degree to which a participant was not feeling deprived, self-conscious, or inconvenienced while engaging with the diet. The “planning and preparation” domain consisted of 5 questions related to how little time was spent on diet application. Scores for each domain were averaged, and acceptability of the intervention was defined as ≥3 for each domain.

#### 2.5.3. Participant Safety

Multiple methods were assessed to monitor safety. Participants completed a standardized adverse events questionnaire via remote survey each week prior to the one-on-one session with the registered dietitian or during the session under the guidance of the registered dietitian. Participants were asked to report incidents of bad breath, headache, nausea, vomiting, weakness, fatigue, irritability, or unhappiness that may be a result of the intervention versus chronic symptoms they were experiencing prior to the start of the intervention [45]. The presence of an event would be noted per week for each participant. Gastrointestinal symptoms of “Constipation,” “Diarrhea,” and “Gas and Bloating” were measured using the PROMIS^®^ (Patient-Reported Outcomes Measurement Information System) Gastrointestinal Symptom Scales [46], which measures symptoms for the previous 7 days. Surveys were administered at baseline and immediately following the intervention. Scores were based on T scores, with an aggregate score of “50” representing the symptomatology of the average population. Lower scores indicate fewer “symptoms”, while higher scores indicate more “symptoms.” Participants were also able to contact the study coordinator at any time throughout the intervention if they believed the diet was resulting in safety concerns.

#### 2.5.4. Body Weight, Body Composition, and Energy Expenditure

Weight was measured between the hours of 6:00 and 10:00 am. Participants were weighed barefoot in light clothing using a balance beam scale (HealthOMeter, Boca Raton, FL, USA) to the nearest 0.25 kg. Scales were provided to participants to monitor weekly weight at home. DXA was used to assess body fat (kilograms), lean mass (kilograms), percent body fat, and visceral fat mass (grams). DXA scans were completed after a 12-h fast between the hours of 6:00 and 10:00 am at study visits. Height was measured at screening using a fixed stadiometer (Seca, Chino, CA, USA). BMI was calculated as weight (kg)/height (m^2^) from baseline height and measured body weight.

Total energy expenditure (TEE) was measured using the Microlife BodyGem^TM^ (Microlife AG, Widnau, Switzerland) resting metabolic rate (RMR) indirect calorimeter [47] after a twelve-hour fast. Participants remained rested for at least 20 min prior to the measurement. The final TEE was calculated by applying an activity rate of 1.2 (“sedentary or light exercise”) to the RMR obtained from the BodyGem^TM^.

#### 2.5.5. Dietary Intake

Participants kept a detailed 7-day food record at baseline and week 12 to determine dietary intake and to determine if any changes were made during the intervention period. A registered dietitian educated each participant on how to accurately report daily intake using a mobile app (Cronometer, Revelstoke, BC, Canada) or on a paper food record. The dietitian verbally validated the food log at the in-person baseline and final data collection visits. This data was entered into the Nutrition Data System for Research (NDSR; Nutrition Coordinating Center, University of Minnesota, Minneapolis, MS, USA) and analyzed for calorie and macronutrient content. Macronutrient data is presented as % of total kcal or g/mg per 1000 kcal.

#### 2.5.6. Physical Activity

Participants were instructed to maintain current levels of physical activity. Physical activity was monitored via FitBit to determine changes in step count over the course of the intervention as a proxy for physical activity. Participants wore the monitor on their wrist for 7 days at baseline and post-intervention, with results analyzed via the Fitabase platform [48].

#### 2.5.7. Circulating Biomarkers

Circulating blood was collected from the antecubital vein of the participants, following a 12 h fast, at baseline and after week 12. Serum glucose, insulin, hemoglobin A1C (HbA1c from whole blood), total, LDL, and high-density lipoprotein (HDL) cholesterol, triglycerides, and high-sensitivity C-reactive protein (hsCRP) were measured by a commercial lab (Quest Diagnostics, Wood Dale, IL, USA). HOMA-IR (Homeostasis Model Assessment for insulin resistance: [HOMA-IR = Fasting insulin (µlU/mL) × Fasting glucose (mg/dL)/405]) was calculated to estimate insulin resistance.

#### 2.5.8. Blood Pressure

Blood pressure was assessed in duplicate with the participant in a seated position after a 10-min rest and with a 5-min rest between measurements using an automatic sphygmomanometer (Omron, Kyoto, Japan).

### 2.6. Power and Sample Size

This pilot study was designed to evaluate the feasibility and acceptability of the TRE and PreTRE interventions and was not powered to detect statistically significant changes in any of the secondary/exploratory measures. The goal was to recruit 20 young adult PCSs participants, allowing for 10% attrition and yielding an expected 18 completers for analysis. With at least 18 completers, we can state with 95% confidence that the trial is feasible, based on the predefined feasibility criterion of an 80% uptake rate. A sample size of 9 participants per group provides at least 80% power to detect a minimum effect size of 1.2 in paired differences from baseline to post-intervention for each primary outcome, using a two-sided paired t-test with a significance level of 0.05. Because this is an exploratory pilot study, power considerations were not applied to overall or multiple pairwise group comparisons, and no type I error correction was made for multiple tests.

### 2.7. Data Management

Data was recorded directly or later uploaded into REDCap [39] (Research Electronic Data Capture Vanderbilt University), a secure web-based platform for building and managing research-related databases and surveys.

### 2.8. Statistical Analysis

Feasibility, acceptability, and secondary data points were summarized using descriptive statistics, including frequencies, percentages, means, standard deviations, and corresponding 95% confidence intervals. For feasibility, we examined recruitment rate among eligible persons that were approached, adherence to the interventions, and percent retained through the end of the intervention. For acceptability, we examined scores on the Diet Satisfaction Survey [44]. An exact binomial test was used to examine whether feasibility and acceptability were significantly higher than their corresponding threshold rates.

For effect size estimation, body weight, body composition, and cardiometabolic markers were summarized as standardized mean changes over time by study group, with corresponding 95% confidence intervals and standard deviations. Within-group changes from baseline to post-intervention were tested using paired *t*-tests when data were normally distributed, with effect sizes estimated by Cohen’s *d*; Wilcoxon signed-rank tests were applied otherwise. Between-group comparisons were conducted using two-sample *t*-tests for normally distributed continuous variables and Wilcoxon rank-sum tests otherwise. Differences in categorical variables between groups were assessed using Fisher’s exact test or chi-square tests. For multivariable analyses, generalized linear models were applied to continuous outcomes and logistic regression models to binary or categorical outcomes, adjusting for age. Age was included as a covariate because it differed between groups at baseline and may influence many secondary outcomes (e.g., weight, cardiometabolic risk markers). All analyses were performed using SAS 9.4 (SAS Institute, Cary, NC, USA).

## 3. Results

### 3.1. Recruitment Feasibility

**Figure 2:**
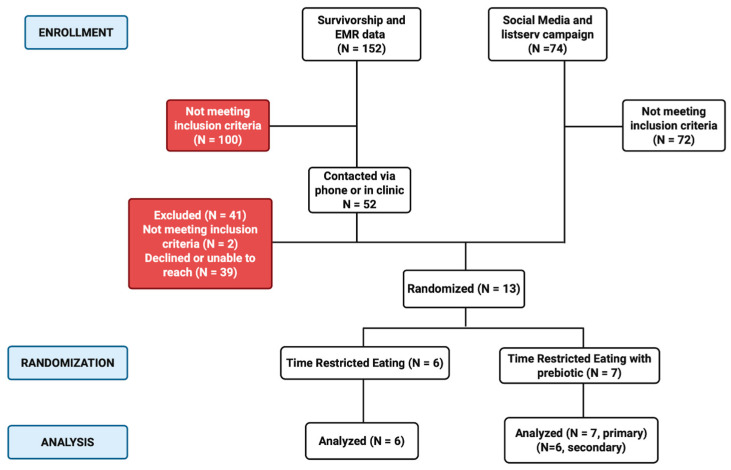
CONSORT (Consolidated Standards of Reporting Trials) flow diagram.

The Childhood Cancer Survivorship Program at UI Health, along with an EMR search, identified 152 potentially eligible patients based on their medical history of surviving a childhood cancer (Figure 2). After additional screening, 100 of those initially identified did not meet the eligibility criteria. The 52 remaining patients were contacted via text message, phone call, email, and/or in person via a clinical visit at UI Health (December 2023–January 2025). Of those contacted, 41 were excluded (*n* = 2 did not meet eligibility criteria; *n* = 3 declined to participate; and *n* = 36 were unable to be contacted). Of the three that declined, two cited not having the time to participate due to life constraints (family responsibilities, change in employment or housing), and one declined due to having previous negative experience with fasting. Eleven of the participants identified through the UI survivorship clinic consented and enrolled in the study.

An additional 74 individuals completed the remote screening survey that was provided via flier, social media campaign, and listserv email. Seventy-two of those individuals were ineligible based on the initial screening tool. Two of these individuals consented and enrolled in the study. A total of 13 eligible participants (24.1%) consented and were randomized into the intervention, which did not meet our a priori hypothesis of 30% (Table 1).

### 3.2. Participant Characteristics

Baseline participant characteristics are described in Table 2. The mean age was 32 (SD 5.34) years. Most participants (*n* = 9, 69%) had a history of blood-based cancers. All the participants had a minimum of some college education, with over half (*n* = 8) having completed a college education or holding graduate or professional degrees. About half of the participants (*n* = 7) were female, and there was equal distribution of racial breakdown, with 3 (23.1%) identifying as non-Hispanic white, 4 (30.8%) identifying as non-Hispanic Black, 4 (30.8%) identifying as Hispanic, and 2 (15.3%) identifying as a racial ethnicity other than non-Hispanic white, non-Hispanic Black or Hispanic.

### 3.3. Attrition

Of the thirteen participants that consented and enrolled, 100% completed the 12-week intervention, indicating a 100% retention rate, which exceeded our a priori hypothesis of 80%. However, one participant in the PreTRE was found to have cancer recurrence approximately 1 month following the completion of the intervention, and data related to secondary outcomes was not included in the final analysis.

### 3.4. Adherence to the Interventions

#### 3.4.1. TRE Window

In the TRE group, the mean eating window per day was 459.1 min (SD 23.3) (7 h and 39 min), while the mean in the PreTRE group was 447.6 min (SD 22.4) (7 h and 28 min) (Table 3). There was no difference between study groups in the average eating window for the 12-week duration. However, there was a significant difference in average minutes per day in weeks 11 and 12 between the TRE group and the PreTRE group (Table 3). The PreTRE group reported a shorter average eating window both weeks, 443.6 min (SD 27.8) (7 h and 24 min) and 436.2 min (SD 44.9) (7 h and 16 min), respectively, while the TRE group reported a longer eating window (491.5 SD 14.4) (8 h and 12 min) (*p* = 0.02) and 497.3 min (SD 35.6) (8 h and 17 min) (*p* = 0.03). Additionally, when examining adherence to the beginning and end of the eating window, both the TRE and the PreTRE had similar adherence (per average days of the week) to the beginning of the eating window, but the PreTRE group was able to adhere to the end of the eating window on more days compared to the TRE group (*p* = 0.04) (Table 4). Adherence to the TRE window was greater than our a priori hypothesis of 80% (Table 1).

#### 3.4.2. Weekly Zoom Sessions

The average number of virtual intervention meetings attended by the TRE group was 11 (SD 1.1), with the total percentage of intervention meetings attended by all participants equal to 87.5%. The average number of virtual intervention meetings attended by the PreTRE group was 11.00 (SD 1.0), with the total percentage of intervention meetings attended by all participants equal to 91.7% (Table 4). There was no difference in session attendance between the two groups. Adherence to the weekly intervention meetings was greater than our a priori hypothesis of 80% (Table 1).

#### 3.4.3. Prebiotic Adherence

The average intake of the prebiotic supplement was 73 (SD 10.6) sachets, which equated to an 87.3% adherence rate (Table 4). Adherence to the prebiotic intake was greater than our a priori hypothesis of 80% (Table 1).

#### 3.4.4. Feasibility of Biospecimen Collection

While all 13 participants successfully completed the intervention, blood collection was not completed for all participants. One participant in the PreTRE group was not included in the analysis due to cancer recurrence post-intervention. In the TRE group, two participants had incomplete biospecimen collection due to the inability of the study team to draw an adequate amount of blood for full analysis.

#### 3.4.5. Acceptability of the Intervention

The TRE and PreTRE groups had similar scores on the Diet Satisfaction Survey at baseline (Table 5). Participants in the TRE group had an increase in scores across all seven domains of the Diet Satisfaction Survey at post-intervention. Participants in the PreTRE group had an increase in the “healthy lifestyle,” “cost,” “family dynamics,” and “planning and preparation” domains of the survey and a decline in the domains of “convenience,” “preoccupation with food,” and “negative aspects” at post-intervention. Both the TRE and the PreTRE group had a greater than “neutral” aggregate score (≥3) in all seven domains of the survey, which met the a priori hypothesis that the interventions would result in an aggregate score of ≥3 on the Diet Satisfaction Survey. Additionally, there was a significant difference in the change in the domain of “preoccupation with food” between the TRE group and the PreTRE group, with the PreTRE group exhibiting a greater decrease in this category following the intervention (*p* = 0.04).

Participants in both groups were asked if they would continue the interventions at the conclusion of the intervention. Four of six participants in the TRE group said they would continue TRE, and four of six participants in the PreTRE group said they would continue TRE with the prebiotic, while one of the six participants would continue the prebiotic only.

#### 3.4.6. Participant Safety

In the TRE group, four participants experienced at least one adverse event, and two participants experienced no adverse events. There were no reported episodes of “bad breath”, “weakness”, or “unhappiness” by any of the TRE participants. “Vomiting”, “headache”, “fatigue”, and “irritability” were reported one time each. “Headache” was reported two times. “Nausea” and “dry mouth” were reported three times.

In the PreTRE group, three (50%) participants experienced at least one adverse event, while three (50%) participants did not report any adverse events throughout the intervention. There were no reported episodes of “vomiting” or “bad breath”. “Dry mouth”, “irritability”, and “unhappiness” were reported one time. “Weakness” was reported two times. “Nausea”, “dizziness”, and “fatigue” were reported three times. “Headache” was reported four times.

GI symptoms were assessed at baseline and post-intervention using the PROMIS^®^ [46] survey. The scores for GI symptoms were similar for TRE and the PreTRE group at baseline (Table 6). Participants in the TRE group had a decrease in “constipation” and “gas and bloating” symptoms and an increase in “diarrhea” symptoms from baseline to post-intervention. The PreTRE group had a decrease in “constipation,” “diarrhea,” and “gas and bloating” symptoms from baseline to post-intervention. There were no differences in the change in symptomology between groups in any of the GI symptom domains at post-intervention.

#### 3.4.7. Cardiometabolic Disease Risk Markers

Table 7 describes the detailed outcomes related to cardiometabolic disease risk markers in the TRE group and PreTRE group.

#### 3.4.8. Body Weight and BMI

BMI, body weight, and body composition were similar in the TRE and PreTRE groups at baseline (Table 2). Participants in the TRE group significantly decreased body weight (mean −4.1 kg; 95% CI [−6.4, −1.8 kg]; t(5) = 4.46; *p* = 0.006) and BMI (mean −1.6 kg/m^2^; 95% CI [−2.5, −0.7 kg/m^2^]; t(5) = 4.46, *p* = 0.007) from baseline to post-intervention. The PreTRE group also decreased body weight and BMI, but within-group changes were not significant. There was a significantly greater decrease in body weight and BMI post-intervention in the TRE group versus the PreTRE group after adjusting for age (body weight: 95% CI [−0.8 to −5.5 kg]; *p* = 0.038; r^2^ = 0.45; BMI: 95% CI [−0.05, −2.26 kg/m^2^]; *p* = 0.042; r^2^ = 0.40).

#### 3.4.9. Body Composition

Fat mass, visceral fat mass, lean mass, and body fat percentage were similar in the TRE group and the PreTRE group at baseline. Participants in the TRE group had a significant decrease in fat mass (mean −2.5 kg; 95% CI [−4.3, −0.7 kg]; t(5) = 3.58; *p* = 0.016) and visceral fat mass (mean −240.5 g; 95% CI [−397.9, −83.1 g]; t(5) = 3.93, *p* = 0.011) from baseline to post-intervention. Participants in the TRE group also decreased lean mass and body fat percentage from baseline to post-intervention, but the decreases were not statistically significant.

Participants in the PreTRE group had a significant decrease in fat mass (mean −2.3 kg; 95% CI [−4.0, −0.7 kg]; t(5) = 3.65; *p* = 0.015) and visceral fat mass (mean −198.3 g; 95% CI [−398.7, −2.0 g]; t(5) = 2.54; *p* = 0.031) from baseline to post-intervention. Participants in the PreTRE group also experienced decreased lean mass and body fat percentage from baseline to post-intervention, although the change was not statistically significant. There was no statistically significant difference between groups regarding body composition changes from baseline to post-intervention.

#### 3.4.10. Circulating Biomarkers

Serum glucose, insulin, HbA1c, triglycerides, HDL, LDL, and total cholesterol were similar between the TRE and PreTRE groups at baseline (Table 7). Participants in the TRE group had a decrease in serum total cholesterol, LDL, HDL, hsCRP, fasting glucose, fasting insulin, and HOMA-IR from baseline to post-intervention, although the changes were not statistically significant. Surprisingly, participants in the TRE group had an increase in triglycerides and HbA1c from baseline to post-intervention, although the change was not statistically significant.

Participants in the PreTRE group had a statistically significant decrease in fasting insulin (mean –3.0 uIU/mL; 95% CI [−5.3, −0.8 uIU/mL]; t(5) = 3.42, *p* = 0.019) from baseline to post-intervention. Total cholesterol, LDL cholesterol, triglycerides, hs-CRP, HbA1c, and HOMA-IR decreased from baseline to post-intervention in the PreTRE group, although the results were not statistically significant. Unexpectedly, participants in the PreTRE group had an increase in fasting glucose from baseline to post-intervention (although these changes were not statistically significant), whilst HDL cholesterol did not change. No differences between groups were observed post-intervention.

#### 3.4.11. Blood Pressure

Systolic and diastolic blood pressure was similar in the TRE and PreTRE groups at baseline. Participants in both the TRE and PreTRE groups had a decrease in systolic and diastolic blood pressure from baseline to post-intervention. However, the reductions in systolic and diastolic blood pressure within each group were not statistically significant. Also, whilst the decreases appeared greater for the PreTRE compared to the decrease in the TRE group, the change in systolic and diastolic blood pressure was not different between groups following the intervention.

#### 3.4.12. Estimated Energy Expenditure and Daily Step Count

Estimated TEE was similar in the TRE and PreTRE groups at baseline. Both groups had an increase in TEE following the intervention (Table 8). There was no statistically significant difference in the change in TEE from baseline to post-intervention between groups.

Average daily step count was used as a proxy for physical activity. Step count was similar in the TRE and PreTRE groups at baseline. Step count increased in the TRE and decreased in the PreTRE group from baseline to post-intervention, but the change was not statistically significant (Table 8).

#### 3.4.13. Self-Reported Calorie Intake

Self-reported caloric intake was similar in the TRE and PreTRE groups at baseline (Table 8). Participants in the TRE groups had an increase in self-reported calorie intake from baseline to post-intervention, although the increase was not statistically significant. Participants in the PreTRE group had a significant decrease in self-reported calorie intake from baseline to post-intervention (mean −450 kcal; 95% CI [−110, −526 kcal]; t(5) = 5.49; *p =* 0.005). We did observe a statistically significant difference in the change in self-reported calorie intake between groups (reflecting the increase in the TRE group and decrease in the PreTRE group) after controlling for age (95% CI −1077.48, −279.0 calories; *p* = 0.006; r^2^ = 0.75).

#### 3.4.14. Self-Reported Dietary Intake

The percent of calories from carbohydrates, fat, and protein and grams of fiber per 1000 kcal were similar in the TRE and PreTRE groups at baseline (Table 8). Participants in the TRE group had an increase in % of calories from protein and a decrease in % of calories from carbohydrates and grams of fiber per 1000 kcal with no change observed in % of calories from fat from baseline to post-intervention (Table 8), although the change was not statistically significant. Participants in the PreTRE group had an increase in % of calories from carbohydrates, protein, and grams of fiber per 1000 kcal and a decrease in % of calories from fat from baseline to post-intervention, although the change was not statistically significant. There was no statistically significant difference between groups in the change of these dietary factors from baseline to post-intervention.

## 4. Discussion

PCSs are burdened by early-onset cardiometabolic disease, which puts them at risk for a compromised quality of life and early mortality. Dietary changes have the potential to ameliorate cardiometabolic disease risk in this high-risk population. This study was, to our knowledge, the first to examine TRE with and without a prebiotic dietary supplement among young adult PCSs as well as the first to combine both TRE and prebiotic supplementation in any healthy or diseased population. We found that the interventions were acceptable, but recruitment of PCSs was not feasible at a single institution. However, retention and adherence to the interventions were high, and there were no major safety concerns given the limited reports of adverse events and the favorable GI symptomatology following the interventions.

While lifestyle interventions may be beneficial for PCSs and reduce the risk of chronic diseases, to our knowledge there are only a handful of published dietary interventions in PCSs with limited success regarding recruitment, retention, and compliance. In Quidde et al., 16 PCSs enrolled in a 4-week hybrid diet intervention (in-person and telephone counseling with a registered dietitian) [49] to improve diet quality based on government guidelines [49]. Participants were counseled to meet an intake of five servings of vegetables and at least 30 g of fiber a day and limit fat and meat consumption. Recruitment rates were not reported; however, of those enrolled, only 70% completed the intervention, and 70% were adherent to the intervention [49]. Another trial enrolled 274 PCSs in a fully remote diet and exercise intervention consisting of a ≥5% daily calorie restriction and ≥180 min of weekly exercise [50]. Only 14.9% (*n* = 41) of participants were ≥ 80% compliant with the interventions [50]. Krull et al. conducted a 24-week in-person exercise program with a daily protein supplement [51]. Participants in this study were recruited from the St. Jude Lifetime Cohort [51]. Sixty-seven PCSs enrolled in the study; 80% were retained for the duration of the trial; and 85% (*n* = 57 participants) were adherent to the intervention >50% of the time [51].

Although our study was small, our retention and intervention adherence metrics suggest that TRE and TRE with a prebiotic supplement are potentially salient interventions among PCSs. Our outcomes related to retention and adherence are reflected in other feasibility studies that employ a TRE regimen in cancer survivors [52,53]. Kirkham et al. reported 98% adherence to an 8-h TRE window among older breast cancer survivors with 100% retention over 8 weeks [52]. A cohort of primarily breast cancer survivors (89.7%) were found to be over 80% adherent to a ten-hour eating window over a period of two weeks with a 92.3% retention rate [53]. Despite the concordance in some feasibility outcomes, these TRE feasibility studies have been more successful in meeting recruitment goals [52,53]. However, the demographics of participants in these studies are different than young adult PCSs. This indicates that more traditional methods of recruitment into this type of lifestyle intervention, such as mailings, may be effective for older (60+ years) survivors of an adult cancer.

A recent review by Wang et al. examined recruitment strategies of adolescent and young adult cancer survivors across 14 trials promoting a behavioral lifestyle intervention [54]. Findings indicated that use of internet-based recruitment strategies, particularly relating to advertisements on social media websites, was most successful in recruiting this survivor population [54]. While our study utilized advertisements on social media, it was limited to Facebook and Instagram, which may not be the social media platform of choice for the age group of interest. While in-person clinical recruitment at a single pediatric oncology clinic in our study was successful, recruitment was limited by a small patient population. Similarly, Rabin et al. was only able to recruit 13 young adult PCSs over a 12-month period via in-person clinical visits at a single oncology clinic [55]. It is possible that even including multiple clinical sites still may not yield the required numbers needed for a clinical trial in young adult PCSs. Cantrell et al. recruited participants in three oncology clinics with active survivorship programs and yet still failed to meet the recruitment requirements needed to power the trial [56]. In-person recruitment leads to better enrollment but fewer numbers, while online strategies yield greater recruitment numbers. Recruitment strategies that employ both methods deployed on a national scale may be critical for conducting lifestyle intervention research in young adult PCSs.

Barriers to recruitment in young adult PCSs include “lack of time” and “lack of perceived benefit” [37,57,58]. Treewek et al. found that providing more information to potential participants regarding time commitment and potential benefits can enhance enrollment [59]. Rabin et al. found that young adult PCSs would be more likely to engage in an intervention trial if the intervention was “convenient” and “provided social support” [60]. Our study may have had successful retention rates due to the convenience of the remote interventions that could be easily tailored to the participant’s schedule as well as the support received during weekly calls from the registered dietitian. Communicating these study features may be important in the initial recruitment process to enhance enrollment.

We explored secondary outcomes related to BMI, body weight, body composition, and cardiometabolic disease risk markers. We observed improvements in body weight and BMI in both groups that were similar to other 8-h TRE interventions conducted in non-cancer populations [23,61,62]. However, we did not see an additional weight loss benefit of the prebiotic when added to TRE, although this could be due to our limited sample size. While there are no other trials combining TRE with a prebiotic, Iversen et al. combined daily calorie restriction with a prebiotic (high-fiber rye wheat) and found no additional weight loss benefit with the addition of the prebiotic [63]. In our trial, whilst no between-group difference in fat mass and visceral fat mass was observed, both the TRE and PreTRE groups had a significant within-group decrease in these parameters from baseline to post-intervention, which may be more impactful than a decrease in BMI/body weight in reducing cardiometabolic disease risk in PCSs. Notably, PCSs have a lower percentage of lean mass to fat mass than the general population, so interventions that reduce fat mass are particularly beneficial to this survivor population [64,65].

Both study groups had a loss of lean body mass, but the PreTRE group lost less lean mass compared to the TRE group. This was somewhat surprising given the TRE group self-reporting a higher % of kcal as protein and increasing their physical activity (assessed via step count) during the intervention. It is possible that the prebiotic supplement supports lean muscle mass due to increases in circulating short-chain fatty acids (SCFAs) such as butyrate, which has epigenetic properties that beneficially impact muscle metabolism [66]. This hypothesis should be explored in future trials [67,68].

Both TRE and PreTRE had improvements in blood pressure and hsCRP following the interventions. The PreTRE group presented slightly greater decreases in blood pressure and hsCRP at post-intervention. Metabolites such as SCFAs produced by beneficial microbes in the context of prebiotic supplementation can activate G protein-coupled receptors and olfactory receptors, which impact pathways to lower blood pressure [69,70,71]. Other studies have shown that prebiotic supplementation can lower circulating hsCRP [72,73]. Butyrate, a type of SCFA, is the preferred energy source of colonocytes [74]. Enhancing the integrity of the gut lining with increased availability of butyrate may prevent another type of microbial metabolite, lipopolysaccharide, from entering the bloodstream [74]. Lipopolysaccharide is an endotoxin, and decreased circulation of this metabolite may decrease systemic inflammation measured by hsCRP [75].

The TRE group had an increase in triglycerides, while the PreTRE group had a decrease in triglycerides at pre-post intervention. TRE interventions have had variable effects on blood lipid levels. Several studies employing an 8-h TRE window found an increase in triglyceride levels among the participants despite concurrent weight loss [23,76]. Studies indicate that triglycerides may increase after prolonged fasting due to the mobilization of lipolysis in fat and muscle tissue with downstream repackaging of fatty acids into triglycerides in the liver [77,78,79]. However, the increase in this lipid level may also be a function of the decrease in HDL, as triglycerides are negatively correlated with levels of HDL in the bloodstream [80]. The TRE group had a decrease in HDL, while the PreTRE group had no change in this marker following the intervention. It is also possible that prebiotic supplementation had a positive impact on lipid metabolism. An increase in circulating SCFAs promoted by prebiotic intake can foster epigenetic modification in liver tissue, downregulating lipogenesis [81], as well as increasing beta oxidation of fatty acids. This would decrease the availability of these molecules to be stored as triglycerides [82].

Both the TRE and PreTRE groups experienced improvements in glucose homeostasis (HOMA-IR). TRE interventions implementing an 8-h eating window for 12 weeks reported similar changes [19,23,61]. Weight loss, particularly visceral fat loss, which both the TRE and PreTRE groups experienced, can improve insulin sensitivity, as visceral fat may contribute to interference in insulin signaling in the liver by the release of free fatty acids [83]. It is likely that a higher degree of body fat loss is needed to observe a clinically relevant effect on glucose homeostasis, although even 12 months of 8-h TRE have failed to make significant changes in these markers [84]. Both TRE and PreTRE groups had a decrease in fasting insulin at post-intervention; however, the PreTRE group saw a greater reduction. The impact of the prebiotic supplement may have affected fasting insulin indirectly via changes in lipid metabolism discussed earlier.

Shortening the daily eating window can result in natural caloric restriction; however, most of the TRE trials have used self-reported estimates of daily calorie intake [23,85]. In this study, the TRE group reported a small (non-significant) increase in caloric intake from baseline to post-intervention, while the PreTRE group reported a significant decrease of about 450 kcal per day, which is similar to other studies following an 8-h TRE window [23,85], and the pre-post intervention change in calorie intake between the two groups was significantly different when controlling for age. Because dietary intake was only assessed at the start and end of the intervention, it is unclear how calorie intake fluctuated day to day or even week to week. It is important to highlight that the PreTRE group reported a significantly shorter eating window in weeks 11 and 12 of the intervention compared to the TRE group, which may have contributed to the calorie decrease observed in their final food log. We hypothesize that the prebiotic may have changed the composition and function of the gut microbiome with effects on satiety, although this hypothesis needs additional follow-up. Future study designs implementing a similar protocol of TRE with and without a prebiotic should consider using doubly labeled water as a gold standard method to understand the effect of energy balance within the context of this intervention.

There are several strengths to this study. This is the first time either TRE or a prebiotic supplement has been explored in young adult PCSs. The interventions were delivered remotely, with participants having access to a registered dietitian via weekly Zoom meetings with appointments available outside of work hours to accommodate a younger age population. Additionally, technology was employed in the use of a food logging app (Cronometer) and text messaging for intervention compliance (TRE window and prebiotic use). Finally, despite the small sample size, the participant group was diverse in terms of race and ethnicity, with as many participants identifying as non-Hispanic Black or Hispanic as non-Hispanic white. This diversity has not been previously captured in dietary lifestyle studies of PCSs.

There were several limitations to this study; most notable is the small sample size given our limited recruitment capabilities at a single site. No control group was included in the analysis, which limits the ability to draw accurate conclusions. Weekly meetings with a registered dietitian were a strength of the study as they pertain to participant engagement, retention, and compliance, yet they may limit the ability of this type of intervention to be readily implemented across all healthcare settings. Dietary data relied on self-report, which has considerable limitations related to response bias with overweight individuals underreporting intake as well as the Hawthorne effect, in which individuals tend to change eating habits due to the knowledge of being monitored [86,87]. Finally, the participants in this study were heterogeneous in terms of cancer type and baseline BMI, and the small sample size prohibited a sub-analysis to stratify for those differences.

### Future Directions

Our study showed high adherence to TRE with or without a prebiotic supplement among PCSs and promising improvements in cardiometabolic disease risk markers. Our data justifies conducting a larger trial in PCSs to determine the clinical application of this type of dietary intervention for the prevention of chronic disease in this high-risk survivor population. Because the methodology of recruitment employed in this study did not meet the sample size goals, modifications in the recruitment strategy would be necessary. A stronger internet-based approach with advertisements across a wide variety of social media sites could potentially enhance recruitment numbers. A regional or even nationwide fully remote intervention would be feasible, with blood-based outcomes collected via home kits [88] or having participants visit a local Quest^®^ Diagnostics (https://www.questhealth.com/ (accessed on 11 June 2025)) location, which are available widely throughout the United States. Body composition outcomes could be collected in a similar way, with participants scheduled to undergo a DEXA scan at one of many facilities, such as Dexafit (https://www.dexafit.com/ (accessed on 11 June 2025)) located in many areas of the United States, or even a body composition home scale. Multiple survivorship clinics could then be targeted for the recruitment of participants, increasing the pool of eligible PCSs. Additionally, a longer intervention period may increase the chance of observing significant improvements in clinical outcomes. Of the participants that completed this study, 61.5% confirmed that they would continue with the dietary regimens, which indicates that compliance may continue past the 12 weeks of intervention implemented in this study.

## 5. Conclusions

The use of TRE with and without prebiotics in young adult PCSs was an acceptable intervention and shows promise for the long-term prevention of chronic disease, although cautionary interpretation of the outcomes of this study is warranted due to the small sample size and relatively short study duration. With high compliance and acceptability of these dietary interventions, TRE and prebiotics may provide an accessible and salient option for PCSs to address the cardiometabolic disease risk that is prevalent in this population. A larger and longer trial, including multiple clinics, is warranted.

## Figures and Tables

**Figure 1:**
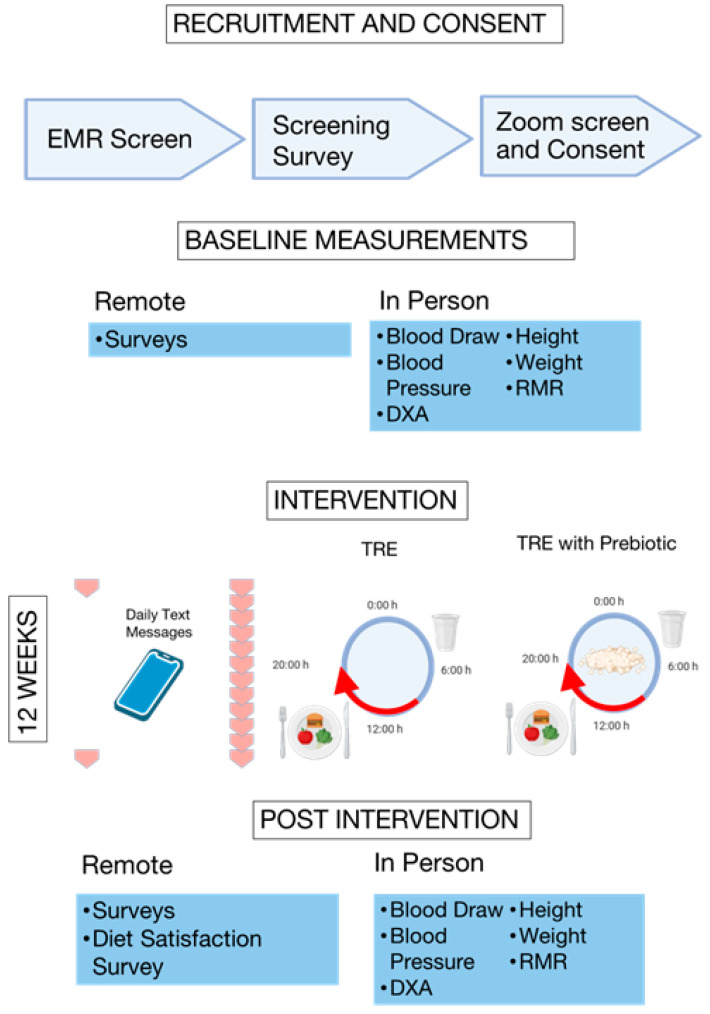
Study design. Design consisted of four distinct time points: recruitment and consent, baseline measurement, intervention and post intervention measurement. The intervention period was 12 weeks which consisted of two data collection time points, daily text messages and weekly meetings with a registered dietitian. Participants would follow the protocol of the group assigned. TRE group engaged in time-restricted eating or ad-libitum caloric intake from 12:00 p.m. to 8:00 p.m. daily. The PreTRE group engaged in time-restricted eating with the addition of daily intake of a prebiotic supplement.

**Table 1:** Feasibility outcomes. The *p* value reflects the exact binomial test between the outcome versus a prior hypothesis, set as a two-tailed outcome for binomial data and a one-sample *t*-test for continuous data.

	A Priori Hypothesis (%)	Mean (%)	
Enrollment (n = 13)	30	24.1	*p* = 0.55
Retention (n = 13)	80	100	*p* = 0.11
Prebiotic Intake (n = 13)	80	87.3	*p* = 0.12
Beginning of Eating Window (n = 12)	80	94.4	*p* < 0.001
End of Eating Window (n = 12)	80	90.6	*p* = 0.001

**Table 2:** Baseline characteristics by intervention group (*n* = 13).

Characteristics	TRE	PreTRE
Participants, *n*	6	7
Mean Age (SD), y	30 (4.8)	33 (5.2)
Female, *n* (%)	3 (50)	5 (71)
Race/ethnic group, *n* (%)		
White	1 (17)	2 (29)
Black	2 (33)	2 (29)
Hispanic	2 (33)	2 (29)
Other or more than one selected	1 (17)	1 (14)
Education, *n* (%)		
Some College	3 (50)	2 (29)
College Graduate	3 (50)	2 (29)
Graduate School or Professional Degree	0 (0)	3 (43)
Cancer Type, *n* (%)		
Leukemia	2 (33)	3 (43)
Lymphoma	2 (33)	2 (29)
Sarcoma	2 (33)	2 (29)
Age at Diagnosis		
Mean Age (SD)	10 (6.4)	16 (9.3)
Body Composition		
Mean Body Weight (SD), kg	98.1 (15.2)	92.5 (13.4)
Mean Height (SD), cm	164.3 (9.6)	168.8 (5.7)
Mean BMI (SD), kg/m^2^	36.2 (4.9)	33.3 (6.6)

Abbreviations: cm—centimeters; kg—kilogram; m—meters; PreTRE—time-restricted eating with prebiotic; SD—standard deviation; TRE—time-restricted eating.

**Table 3:** Mean weekly eating window duration for the TRE group and the PreTRE group.

	TRE			PreTRE			
	*n*	Mean	SD	*n*	Mean	SD	Between-Group
Week 1	6	441.4	50.4	6	447.2	21.5	*p* = 0.62
Week 2	6	458.2	24.5	6	443.5	19.0	*p* = 0.48
Week 3	6	441.2	53.1	6	446.0	52.5	*p* = 0.96
Week 4	6	461.4	39.2	6	455.0	24.3	*p* = 0.92
Week 5	6	443.5	39.1	6	455.4	37.3	*p* = 0.19
Week 6	6	466.6	22.2	6	465.5	19.6	*p* = 0.18
Week 7	6	452.3	33.6	6	443.7	28.6	*p* = 0.68
Week 8	6	465.5	27.1	6	435.3	20.3	*p* = 0.17
Week 9	6	429.4	59.0	6	455.2	44.0	*p* = 0.36
Week 10	6	461.2	48.6	6	444.6	59.2	*p* = 0.39
Week 11	6	491.5 ^‡^	14.4	6	443.6 ^‡^	27.8	*p* = 0.02
Week 12	6	497.3 ^‡^	35.6	6	436.2 ^‡^	44.9	*p* = 0.03
Overall	6	459.1	23.2	6	447.6	22.4	*p* = 0.32

Abbreviations: PreTRE—time-restricted eating with prebiotic; SD—standard deviation; TRE—time-restricted eating. ^‡^ Indicates between-group statistical significance.

**Table 4:** Compliance to the eating window, prebiotic intake and weekly RD meetings.

	TRE				PreTRE				
	*n*	Mean	SD	Percent	*n*	Mean	SD	Percent	Between-Group
Beginning of Eating Window (days per week)	6	6.4	0.4	92.1	6	6.8	0.3	96.7	*p* = 0.24
End of Eating Window (days per week)	6	6.0 ^‡^	0.7	85.3	6	6.7 ^‡^	0.3	95.8	*p* = 0.04
Prebiotic Intake (days)					6	73.3	10.6	87.3	
Weekly intervention sessions	6	11	1.1	87.5	6	11	0.9	91.7	*p* = 0.45

Abbreviations: PreTRE—time-restricted eating with prebiotic; RD—registered dietitian; SD—standard deviation; TRE—time-restricted eating. ^‡^ Indicates between-group statistical significance (set at *p* < 0.05) as measured by a generalized linear model, assuming normality and including “age” as a covariate.

**Table 5:** Diet and satisfaction survey in the TRE group and the PreTRE group with the average score totaled, with scores ranging from “0” to “5”.

	TRE								PreTRE								
		Baseline		Post		Change				Baseline		Post		Change			
Domains	*n*	Mean	SD	Mean	SD	Mean	SD	Within-Group	*n*	Mean	SD	Mean	SD	Mean	SD	Within-Group	Bt-Group
Healthy Lifestyle	6	3.4	0.58	3.8	0.42	0.5	15.0	*p* = 0.02	6	3.0	0.75	3.4	0.35	0.4	0.56	*p* = 0.16	*p* = 0.70
Convenience	6	3.4	0.45	3.7	0.19	0.2	0.40	*p* = 0.23	6	3.6	0.58	3.4	0.51	−0.2	0.30	*p* = 0.23	*p* = 0.08
Cost	6	2.8	0.73	3.0	0.55	0.2	0.50	*p* = 0.45	6	3.1	0.47	3.6	0.59	0.5	0.72	*p* = 0.16	*p* = 0.37
Family Dynamics	6	3.5	0.27	3.7	0.20	0.1	0.36	*p* = 0.48	6	3.5	0.54	3.6	0.33	0.1	0.33	*p* = 0.44	*p* = 1.0
Preoccupation with Food	6	3.3	0.48	3.7	0.38	0.4	0.39	*p* = 0.05	6	3.3	0.96	3.2	0.84	−0.1	0.42	*p* = 0.33	*p* = 0.04
Negative Aspect	6	3.8	0.63	4.1	0.36	0.3	0.77	*p* = 0.41	6	3.9	0.74	3.6	0.87	−0.3	0.70	*p* = 0.42	*p* = 0.84
Planning and Preparation	6	3.0	0.79	3.0	0.63	0.0	0.50	*p* = 0.88	6	3.3	0.65	3.5	0.49	0.2	0.61	*p* = 0.39	*p* = 0.55

Abbreviations: Bt—between; PreTRE—time-restricted eating with prebiotic; SD—standard deviation; TRE—time-restricted eating.

**Table 6:** GI symptom data as measured by the PROMIS^®^ gastrointestinal scale in the TRE group and the PreTRE group.

	TRE (*n* = 6)								PreTRE (*n* = 6)								
	Baseline		Post		Change				Baseline		Post		Change				
	Mean	SD	Mean	SD	Mean	SD	Effect Size	Within-Group	Mean	SD	Mean	SD	Mean	SD	Effect Size	Within-Group	Bt-Group
Constipation	50	7.6	46	7.6	−3	5.3	−0.6	*p* = 0.18	50	7.9	43	4.5	−6	8.4	−0.7	*p* = 0.14	*p* = 0.52
Diarrhea	47	6.8	48	9.6	1	10.2	0.1	*p* = 0.81	42	5.9	41	1.8	−1	6.7	−0.2	*p* = 0.67	*p* = 0.65
Gas and Bloating	51	8.0	44	7.4	−8	6.1	−1.3	*p* = 0.25	53	6.8	45	5.9	−8	8.7	−0.9	*p* = 0.08	*p* = 0.96

Abbreviations: Bt—between; PreTRE—time-restricted eating with prebiotic; SD—standard deviation; TRE—time-restricted eating.

**Table 7:** Cardiometabolic outcomes in the TRE group and the PreTRE group.

	TRE									PreTRE									
		Baseline		Post		Change					Baseline		Post		Change				
	*n*	Mean	SD	Mean	SD	Mean	SD	Effect Size	Within-Group	*n*	Mean	SD	Mean	SD	Mean	SD	Effect Size	Within-Group	Between-Group
Body Composition Markers																			
BW kg	6	98.1	16.7	94.0	17.5	−4.1 ^†‡^	2.2	−1.9	*p* = 0.006	6	92.5	14.6	90.3	13.1	−2.2 ^‡^	2.3	−1.0	*p* = 0.07	*p* = 0.04
BMI	6	36.2	5.3	34.6	5.4	−1.6 ^†‡^	0.9	−1.8	*p* = 0.007	6	33.3	7.2	32.5	6.8	−0.8 ^‡^	0.8	−1.0	*p* = 0.06	*p* = 0.04
Fat Mass kg	6	38.7	13.8	36.2	14.0	−2.5 ^†^	1.7	−1.5	*p* = 0.02	6	39.3	14.5	37.0	13.7	−2.3 ^†^	15.6	−1.5	*p* = 0.02	*p* = 0.77
Lean Mass kg	6	55.4	15.6	54.4	15.0	−1.0	1.2	−0.9	*p* = 0.09	6	50.1	7.2	50.4	7.3	−0.3	17.7	0.2	*p* = 0.73	*p* = 0.08
Visceral g	6	1197.0	499.0	956.5	507.8	−240.5 ^†^	150.0	−1.6	*p* = 0.01	6	1124.7	702.8	926.3	556.2	−198.3 ^†^	190.9	−1.0	*p* = 0.03	*p* = 0.52
Fat %	6	41.2	12.4	40.0	12.4	−1.2	1.4	−0.8	*p* = 0.09	6	42.7	10.1	40.0	11.1	−2.7	3.8	−0.7	*p* = 0.14	*p* = 0.44
Cardiovascular Disease Risk Markers																			
T Chol mg/dL	4 *	182	20.9	172	18.3	−10	11.2	−0.9	*p* = 0.17	6	172	22.1	169	20.4	−2	14.1	−0.2	*p* = 0.70	*p* = 0.35
LDL mg/dL	4 *	116	21.7	104	22.7	−12	2.5	−2.0	*p* = 0.13	6	96	14.8	95	13.4	−2	14.5	−0.1	*p* = 0.85	*p* = 0.22
HDL mg/dL	4 *	49	5.4	48	8.4	−1	5.6	−0.2	*p* = 0.74	6	54	11.7	54	11.4	0	3.2	0	*p* = 1.0	*p* = 0.46
TG mg/dL	4 *	77	15.7	101	18.3	24	16.9	1.4	*p* = 0.06	6	122	66.6	109	67.8	−13	50.9	−0.3	*p* = 0.56	*p* = 0.18
SBP mmHg	6	126	12.4	123	10.4	−3	8.8	−0.4	*p* = 0.43	6	131	7.1	121	7.0	−10	12.3	−0.9	*p* = 0.06	*p* = 0.67
DBP mmHg	6	80	4.6	79	3.7	−1	6.5	−0.1	*p* = 0.57	6	86	5.8	83	5.7	−2	9.7	−0.3	*p* = 0.74	*p* = 0.96
hsCRP mg/L	6	4.9	3.1	4.7	3.6	−0.2	1.6	0.1	*p* = 0.81	6	3.5	2.6	2.7	3.6	−0.7	3.3	−0.2	*p* = 0.62	*p* = 0.39
Glucoregulatory Markers																			
Glucose mg/dL	5 *	92	11.5	87	10.1	−5	6.2	−0.9	*p* = 0.12	6	89	6.7	93.3	5.2	5	9.5	0.5	*p* = 0.28	*p* = 0.14
Insulin uIU/mL	5 *	17.0	12.2	14.0	6.4	−3.0	6.0	−0.5	*p* = 0.33	6	11.8	7.9	8.8	6.4	−3.0 ^†^	2.2	−1.4	*p* = 0.02	*p* = 0.69
HbA1c % of Hgb	6	5.4	0.4	5.5	0.3	0.1	0.2	0.3	*p* = 0.44	6	5.4	0.2	5.4	0.2	−0.03	0.1	−0.3	*p* = 0.53	*p* = 0.42
HOMA-IR	5 *	4.1	3.5	3.1	1.7	−1.0	1.9	−0.6	*p* = 0.28	6	2.6	1.8	2.0	1.5	−0.5	0.6	−0.8	*p* = 0.09	*p* = 0.39

Abbreviations: HbA1c—hemoglobin A1C; BMI—body mass index; BW—body weight; DBP—diastolic blood pressure; g—grams; HDL—high-density lipoprotein; HOMA-IR—Homeostatic Model Assessment for Insulin Resistance; kg—kilograms; LDL—low-density lipoprotein; SD—standard deviation; T Chol—total cholesterol; TG—triglycerides; SBP—systolic blood pressure. * Number of participants with blood at both baseline and post-intervention timepoints; ^†^ Within-group statistical significance; ^‡^ Between-group statistical significance.

**Table 8:** Step count, total energy expenditure, and diet data in the TRE group and the PreTRE group.

	TRE									PreTRE							
		Baseline		Post		Change				Baseline		Post		Change			
	*n*	Mean	SD	Mean	SD	Mean	SD	Within-Group	*n*	Mean	SD	Mean	SD	Mean	SD	Within-Group	Between-Group
Step Count and Total Energy Expenditure																	
Step count	6	9501	3810.5	9414	3340.9	1580	4422.9	*p* = 0.90	6	5897	3798.4	5393	2353.2	−932	2686.8	*p* = 0.74	*p* = 0.06
TEE	6	2363	339.8	2368	396.8	4	172.9	*p* = 0.96	6	2173	202.2	2357	236.3	154	247.7	*p* = 0.22	*p* = 0.28
Diet Data																	
Daily kcal	6	1723	434.9	1847	357.5	124 ^‡^	219.5	*p* = 0.32	6	1841	439.5	1392	488.1	−450 ^†‡^	163.8	*p* = 0.005	*p* = 0.006
% kcal from carbohydrates	6	42.6	6.35	42.2	13.59	−0.4	11.52	*p* = 0.95	6	40.3	13.70	44.9	10.39	4.6	6.15	*p* = 0.21	*p* = 0.47
% kcal from protein	6	19.9	4.87	23.2	7.87	3.2	5.00	*p* = 0.26	6	18.9	4.11	19.4	8.59	0.5	9.56	*p* = 0.91	*p* = 0.63
% kcal from fat	6	37.4	4.32	37.4	7.61	0.0	8.36	*p* = 0.99	6	39.0	9.72	33.3	9.14	−5.7	8.99	*p* = 0.27	*p* = 0.38
Grams fiber per 1000 kcal	6	8.9	1.81	8.6	2.37	−0.4	0.83	*p* = 0.40	6	8.5	1.81	9.2	4.47	0.7	0.40	*p* = 0.06	*p* = 0.07

Abbreviations: kcal—kilocalories; PreTRE—time-restricted eating with prebiotic; SD—standard deviation; TEE—total energy expenditure; TRE—time-restricted eating. ^†^ Within-group statistical significance; ^‡^ Between-group statistical significance.

## Data Availability

Data are unavailable due to privacy or ethical restrictions.

## Abbreviations

The following abbreviations are used in this manuscript:

PCS	Pediatric cancer survivor
TRE	Time-restricted eating
LDL	Low-density lipoprotein
SCFA	Short-chain fatty acid
GOS	Galacto-oligosaccharides
BMI	Body mass index
DXA	Dual X-ray absorptiometry
KG	Kilogram
M	Meter
TEE	Total energy expenditure
RD	Registered dietitian
RMR	Resting metabolic rate
NDSR	Nutrition data system for research
HbA1c	Hemoglobin A1c
HDL	High-density lipoprotein
HOMA-IR	Homeostasis Model Assessment for Insulin Resistance
QUICKI	Quantitative insulin-sensitivity check index
CI	Confidence interval
hsCRP	High-sensitivity C-reactive protein
GLP-1	Glucagon-like peptide 1
PYY	Peptide tyrosine tyrosine
